# Humanizing the Protease-Activated Receptor (PAR) Expression Profile in Mouse Platelets by Knocking *PAR1* into the *Par3* Locus Reveals PAR1 Expression Is Not Tolerated in Mouse Platelets

**DOI:** 10.1371/journal.pone.0165565

**Published:** 2016-10-27

**Authors:** Shauna L. French, Antonia C. Paramitha, Mitchell J. Moon, Ross A. Dickins, Justin R. Hamilton

**Affiliations:** Australian Centre for Blood Diseases, Monash University, Melbourne, Australia; University of Kentucky, UNITED STATES

## Abstract

Anti-platelet drugs are the mainstay of pharmacotherapy for heart attack and stroke prevention, yet improvements are continually sought. Thrombin is the most potent activator of platelets and targeting platelet thrombin receptors (protease-activated receptors; PARs) is an emerging anti-thrombotic approach. Humans express two PARs on their platelets–PAR1 and PAR4. The first PAR1 antagonist was recently approved for clinical use and PAR4 antagonists are in early clinical development. However, pre-clinical studies examining platelet PAR function are challenging because the platelets of non-primates do not accurately reflect the PAR expression profile of human platelets. Mice, for example, express Par3 and Par4. To address this limitation, we aimed to develop a genetically modified mouse that would express the same repertoire of platelet PARs as humans. Here, human *PAR1* preceded by a lox-stop-lox was knocked into the mouse *Par3* locus, and then expressed in a platelet-specific manner (hPAR1-KI mice). Despite correct targeting and the predicted loss of Par3 expression and function in platelets from hPAR1-KI mice, no PAR1 expression or function was detected. Specifically, PAR1 was not detected on the platelet surface nor internally by flow cytometry nor in whole cell lysates by Western blot, while a PAR1-activating peptide failed to induce platelet activation assessed by either aggregation or surface P-selectin expression. Platelets from hPAR1-KI mice did display significantly diminished responsiveness to thrombin stimulation in both assays, consistent with a *Par3-/-* phenotype. In contrast to the observations in hPAR1-KI mouse platelets, the PAR1 construct used here was successfully expressed in HEK293T cells. Together, these data suggest ectopic PAR1 expression is not tolerated in mouse platelets and indicate a different approach is required to develop a small animal model for the purpose of any future preclinical testing of PAR antagonists as anti-platelet drugs.

## Introduction

Anti-platelet drugs are the primary therapy for heart attack and stroke prevention, yet improvements are continually sought. Thrombin is by far the most potent endogenous platelet activator, which it achieves via cell surface protease-activated receptors (PARs). Due to the importance of thrombin-induced platelet activation during thrombosis, targeting platelet thrombin receptors has received significant clinical attention, and PAR antagonists are one of the most promising of the emerging anti-thrombotic approaches [1–3]. Humans express two PARs on their platelets, PAR1 and PAR4, both of which are cleaved and activated by thrombin, and both of which are capable of inducing robust platelet activation [4, 5]. PAR1 has a higher affinity for thrombin and was therefore the target of initial drug development. The first PAR1 antagonist, vorapaxar, was recently approved by the FDA for clinical use in the USA [2]. So far, however, vorapaxar has limited clinical utility due to an increase in major bleeding events associated with its use [6, 7]. These limitations on the first PAR1 inhibitor have ignited interest in evaluating the clinical potential of PAR4 antagonists, and these agents are now in early clinical development (NCT02208882) [8].

However, a major limitation in examining platelet PAR function in detail is the absence of a small animal model that accurately reflects the PAR expression profile in human platelets: only primate platelets express the PAR1/PAR4 profile [9]. This places significant limits on preclinical research into the utility of PAR antagonists *in vivo*. Of the commonly-used small animals, for example, platelets from mice, rats and rabbits express PAR3 and PAR4 [10–13], while guinea-pig platelets express PAR1, PAR3 and PAR4 [14]. *Par4-/-* mice have been crucial for early proof-of-concept studies into the overall role of PARs in thrombosis [15–17], but are unsuitable for elucidating, for example, the relative anti-thrombotic effects of PAR1 vs PAR4 inhibition.

One way to overcome this limitation is to ‘humanize’ the platelet PAR expression profile of the mouse, essentially replacing mouse PAR3 (Par3) with human PAR1 (PAR1). Initial attempts used platelet-specific expression of a PAR1 transgene in *Par3-/-* mice, but were unsuccessful [18]. Possible explanations for the failure of this transgene-based approach include insufficient expression levels or unpredicted gene silencing consequences as a result of nonspecific transgene insertion [18]. We theorized that ‘knock-in’ of PAR1 into the endogenous mouse *Par3* locus in a manner that would allow platelet-specific expression may overcome these earlier issues and would create a mouse that expresses PAR1 and Par4 on platelets, but without further ectopic PAR1 expression. Here, we generate and characterise such a mouse. Despite correct targeting, this genetic approach failed to yield detectable expression or function of PAR1 on mouse platelets. When taken together with earlier studies using distinct approaches, this study indicates that forced expression of PAR1 in mouse platelets will be difficult to achieve and suggests that other options will be required for reliable preclinical screening of any future PAR antagonists.

## Methods and Materials

### Materials

Human α-thrombin and adenosine 5’-diphosphate sodium salt (ADP) were purchased from Sigma-Aldrich (St Louis, MO, USA). PAR1-activating peptide (PAR1-AP; TFLLR-NH_2_) and PAR4-activating peptide (PAR4-AP; AYPGKF-NH_2_) were synthesised at Monash Institute of Pharmaceutical Sciences (Melbourne, Australia) by Assoc Professor Philip Thompson. The mouse anti-P-selectin (FITC anti-mouse CD62P) and human anti-P-selectin (PE anti-human CD62P) antibodies were purchased from BD Biosciences (San Jose, CA, USA). PE anti-CD45.2, PE anti-CD41a (human) antibody and PE anti-CD41 antibody (mouse) were purchased from Abcam (Melbourne, Australia). The PE-anti-GPIb-tail and non-immune rabbit IgG isotype were a generous gift from Assoc Professor Robert Andrews (Monash University, Melbourne, Australia). The anti-PAR1 antibody (ATAP2), anti-PAR3 antibody (8E8), HRP-anti-actin (I-19) and HRP- anti-mouse IgG were purchased from Santa Cruz Biotechnology (Santa Cruz, CA, USA).

### Generation of hPAR1-KI (*Pf4-Cre; Par3*^*LSL-PAR1*^) mice

**Fig 1:**
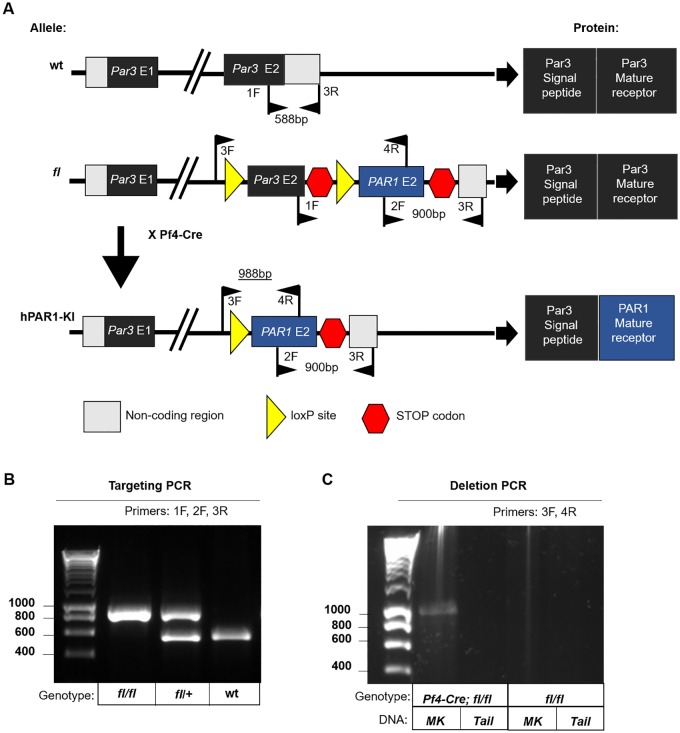
Generation of mice expressing PAR1 in place of Par3 in a platelet-specific manner. **(A)** Schematic of the strategy for platelet-specific expression of human PAR1 in place of mouse Par3. Top: wild-type *Par3* (wt) has two exons: exon 1 encodes the signal peptide and exon 2 encodes the complete mature receptor. Middle: In the targeted allele (*Par3*^LSL-PAR1^; fl) an insert of exon 2 of human *PAR1* is placed downstream of exon 2 of *Par3*. *Par3* exon 2 is flanked by loxP sites, allowing Cre-mediated excision. Bottom: Cre-mediated deletion of *Par3* exon 2 allows expression of *PAR1* exon 2, resulting in a predicted chimeric protein consisting of the Par3 signal peptide fused to the mature PAR1 receptor (hPAR1-KI). Heterozygous targeted mice, *Par3*^LSL-PAR1/+^ (fl/+); homozygous targeted mice, *Par3*^LSL-PAR1/LSL-PAR1^ (fl/fl); platelet-specific replacement of mPAR3 with hPAR1, Pf4-Cre; *Par3*^LSL-PAR1^ (Pf4-Cre;fl/fl). **(B)** PCR genotyping of tail DNA using the primers indicated (black flags): wild type forward (1F), mutant forward (2F), common reverse (3R). **(C)** Cre-mediated deletion of *Par3* was confirmed by PCR genotyping of individual mice using DNA from both tail (not deleted) and megakaryocytes (MK; deleted), using a forward primer spanning the loxP site (3F) and *PAR1* reverse (4R). A PCR product of predicted size (988bp) was only generated using MK DNA from Pf4-Cre;fl/fl mice. Sequencing of this PCR product confirmed excision of floxed sequences (S3 Fig).

All mouse studies were approved by the Alfred Medical Research and Education Precinct Animal Ethics Committee (approval number E/1465/2014/M). *Pf4-Cre; Par3*^*LSL-PAR1*^ mice (hereafter referred to as hPAR1-KI mice) were generated by Ozgene (Perth, Australia). The targeting vector involved exon 2 of *Par3* (*F2rl2*) flanked with a lox-stop-lox and followed by exon 2 of *PAR1 (F2r)* (Fig 1A). Since exon 1 encodes the signal peptide and exon 2 encodes the entire mature receptor for both PAR1 and Par3 [19], Cre-mediated deletion is predicted to result in the replacement of Par3 with PAR1 and leaves the endogenous *Par3* promoter intact (Fig 1A). The BAC vector comprised unaltered exon 1 of *Par3*, then exon 2 of *Par3* followed by a stop codon and flanked by loxP sites, and followed by *PAR1*. The vector was injected into C57BL/6J blastocysts, chimeras were bred for germline transmission, and the targeted allele bred to homozygosity (*Par3*^*LSL-PAR1*^). These mice were then bred with Pf4-Cre mice to induce deletion of *Par3* and permit the expression of *PAR1* specifically in platelets (*Pf4-Cre; Par3*^*LSL-PAR1*^, hereafter referred to as hPAR1-KI mice). PCR genotyping of the targeted allele was performed using the following primers: common reverse (AGCTGAAAAATGGAGCGCTTG) with WT forward (TGGGTTCCTCATCCCGTTTG, predicted size 588bp) or mutant forward (TCCTTCACTTGTCTGGCCATC, predicted size 914bp) (Fig 1A). Deletion of *Par3* was confirmed by PCR using the following primers: forward (CAGTGTGTGGTTTTGTTTCACCT) and reverse (GCCAATCACTGCCGGAAAAG, predicted size 963bp) (Fig 1A).

### Isolation of mouse and human platelets

All human studies were approved by the Monash University Human Research Ethics Committee (CF07/0141-2007/0025). Blood was collected after written informed consent was obtained from healthy adults (21–50 years old, of both sexes) who had not taken anti-platelet medications in the past 10 d. Mouse and human blood was drawn into syringes containing acid citrate dextrose (ACD; 1:7 v/v). Platelets were isolated by centrifugation as previously described [20].

### Megakaryocyte DNA extraction

Bone marrow cells were aseptically flushed from the femora and tibiae of mice using a syringe and a 23-gauge needle containing Dulbecco’s Modified Eagle Medium (DMEM, Life Technologies) supplemented with 10% foetal bovine serum (FBS), penicillin, streptomycin and glutamine. Post-harvest, thrombopoietin (0.1 μg/mL) was added to the cells, which were allowed to culture for 4 d at 37°C, 5% CO_2_. Cells were then collected and centrifuged at 50 g for 5 min and the pellet was re-suspended in 4 mL of PBS. The cells were layered over a two-step BSA density gradient and allowed to sediment for 45 min at room temperature. The megakaryocyte-rich population was collected and centrifuged at 50 g for 5 min, and the pellet was treated with DNA extraction buffer (Bioline) and digested at 75°C for 10 min.

### Platelet aggregation

Platelet aggregation was measured by light transmission aggregometry in a 96-well plate format. Human and mouse washed platelets (2x10^8^ platelets/mL) were added to wells of a clear, flat bottom, 96-well tissue culture plate (Falcon). Platelets were stimulated with either thrombin (0.01–1 U/mL), PAR1-AP (10–100 μM), PAR4-AP (10–100 μM), or ADP (1–10 μM). The plate was analysed at 37°C in a FLUOstar OPTIMA plate reader (BMG Labtech) using a 595 nm excitation filter, for a period of 50 min (10 read cycles with 5 min double orbital shake period between each read). Ability to aggregate was calculated as $\frac{OD\left(No\;Agonist\right)-OD\left(Agonist\right)}{OD\left(No\;Agonist\right)-OD\left(blank\right)}\times 100$ at the time point where aggregation was at a maximum. Optical density was normalised against the blank (maximum) and unstimulated platelets (minimum) and expressed as % maximum.

### P-selectin expression

Flow cytometry was used to detect the expression of P-selectin by activated platelets. Human and mouse platelets, prepared as outlined above, were incubated with an anti-P-selectin antibody and stimulated with thrombin (0.01–1 U/mL), PAR1-AP (10–100 μM), or PAR4-AP (30–300 μM) for 15 min at 37°C. Samples were then analysed using a FACSCalibur (Becton Dickinson) flow cytometer and FlowJo software.

### Analysis of platelet PAR1 expression

Platelets isolated from either humans or mice (5x10^7^ platelets/mL in Tyrode’s buffer) were incubated with a PE-conjugated antibody against one of PAR1 (2.5 μg/10^6^ cells; ATAP2), CD45 (platelet negative control antibody of matching isotype, 2.5 μg/10^6^ cells), or CD41a (platelet positive control antibody of matching isotype, 2.5 μg/10^6^ cells). In some experiments, isolated platelets were permeabilized with saponin (0.1% v/v) prior to incubation with antibodies. In these experiments, a PE-conjugated antibody against the cytoplasmic tail of GPIb was included to confirm effective platelet permeabilization [21]. In all cases, samples were analysed using a FACSCalibur flow cytometer and FlowJo Software. Positive expression was measured as a rightward shift in relative fluorescent intensity compared to the isotype control.

### Western blot

Platelet proteins were analyzed in lysates of human or mouse isolated platelets (1x10^9^ platelets/mL), prepared in Laemmli’s buffer, denatured (95°C for 10 min), and run on 12% SDS-polyacrylamide gels at 170 V for 40 min. Proteins were transferred to PVDF membranes (Millipore, Lake Placid, NY, USA) at 250 mA for 2 h. Membranes were blocked with TBST (0.01 mM Tris, 50 mM NaCl, 0.01% v/v Tween-20) containing 5% skim milk powder for 30 min at room temperature, incubated with either an HRP-conjugated anti-actin antibody (1 μg/mL), anti-PAR1 antibody (1 μg/mL) or anti-PAR3 antibody (1 μg/mL) at 4°C overnight, followed by a HRP-conjugated anti-mouse IgG at room temperature for 2 h. Enhanced chemiluminescence (ECL) substrate (ThermoFisher Scientific, Waltham, MA, USA) was placed onto the membrane for 1 min before exposure (ChemiDoc Touch Imaging System, Bio-Rad, Hercules, CA, USA).

## Results

### Generation of hPAR1-KI (*Pf4-Cre; Par3*^*LSL-PAR1*^) mice

We generated mice in which *PAR1* (*F2R*) was knocked into the *Par3* (*F2lr2*) locus and conditionally expressed in platelets upon Pf4-Cre-mediated excision. To this end, exon 2 of *PAR1* (encoding the entire mature receptor) was inserted downstream of exon 2 of *Par3* flanked by a lox-stop-lox (Fig 1A). This approach leaves the promoter and exon 1 of *Par3* (encoding the signal peptide) intact and results in replacement of *Par3* with *PAR1* after Cre-mediated excision (Fig 1A). This is predicted to yield the Par3 signal peptide linked to the complete mature receptor of PAR1 (Fig 1A). The stability and surface expression of the predicted fusion protein was confirmed in HEK293T cells transiently transfected with vectors expressing the same sequence as that remaining after Cre-mediated excision of the targeted allele, with the predicted Par3/PAR1 fusion protein expressed at similar levels to native PAR1 (S1 Fig). We therefore went on to generate *Par3*^*LSL-PAR1/+*^ mice and confirmed targeting by Southern blot (S2 Fig) and PCR genotyping (Fig 1B). Heterozygous mice were bred to homozygosity (*Par3*^*LSL-PAR1/LSL-PAR1*^) and crossed with Pf4-Cre mice [22], with experimental mice resulting from crosses of *Pf4-Cre;Par3*^*LSL-PAR1/LSL-PAR1*^ x *Par3*^*LSL-PAR1/LSL-PAR1*^ (Fig 1B). Cre positive offspring (*Pf4-Cre;Par3*^*LSL-PAR1/LSL-PAR1*^) are hereafter referred to as hPAR1-KI mice and their Cre negative littermates (*Par3*^*LSL-PAR1/LSL-PAR1*^) as wild-type. Appropriate Pf4-Cre-mediated deletion in the platelet/megakaryocyte lineage was confirmed by PCR of DNA isolated from megakaryocytes versus tail biopsy (Fig 1C). We used a forward primer placed upstream of the loxP site and a reverse primer in each of *Par3* and *PAR1* as indicated (Fig 1A). As predicted, a band of expected size was only generated in megakaryocyte DNA from hPAR1-KI mice. Sequencing of this PCR product confirmed the predicted targeting and Cre-mediated deletion (S3 Fig).

### Platelets from hPAR1-KI mice do not respond to PAR1-selective activation

**Fig 2:**
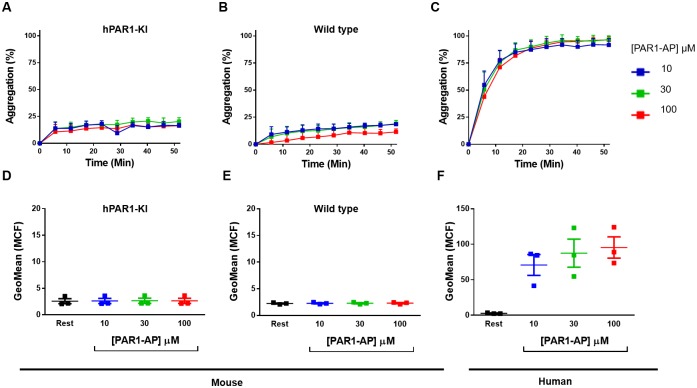
Platelets from hPAR1-KI mice do not respond to PAR1-selective activation. Platelets isolated from hPAR1-KI mice, wild type mice, or humans were stimulated with a PAR1-activating peptide (PAR1-AP; TFLLR) and examined for **(A-C)** platelet aggregation by light transmission aggregometry or **(D-F)** P-selectin expression by flow cytometry. Note that platelets from either hPAR1-KI or wild type mice failed to respond to PAR1-AP, even at concentrations that were supra-maximal in human platelets. Data are mean ± SEM of n = 3 experiments.

Mice from these crosses were born at the expected rates, were normal in weight and appearance, and had normal whole blood counts (S1 Table). We assessed PAR1 function in platelets isolated from hPAR1-KI mice using two distinct markers of platelet activation: platelet aggregation and P-selectin expression. PAR1-selective activation by a PAR1-AP failed to aggregate platelets from hPAR1 mice at concentrations up to 100 μM (100 times the concentration required to cause platelet activation in human platelets [23]) (Fig 2A). This lack of response was identical in platelets from wild type mice (Fig 2B). As expected, PAR1-AP induced a robust aggregation response in human platelets (Fig 2C). A similar pattern of responses was observed when P-selectin expression was used as the marker of platelet activation, with PAR1-AP unable to elicit any response in platelets isolated from either hPAR1-KI (Fig 2D) or wild type (Fig 2E) mice, but a robust response in human isolated platelets (Fig 2F). Together, these data indicate lack of functional PAR1 in platelets from hPAR1-KI mice.

### Lack of PAR1 expression on platelets from hPAR1-KI mice

**Fig 3:**
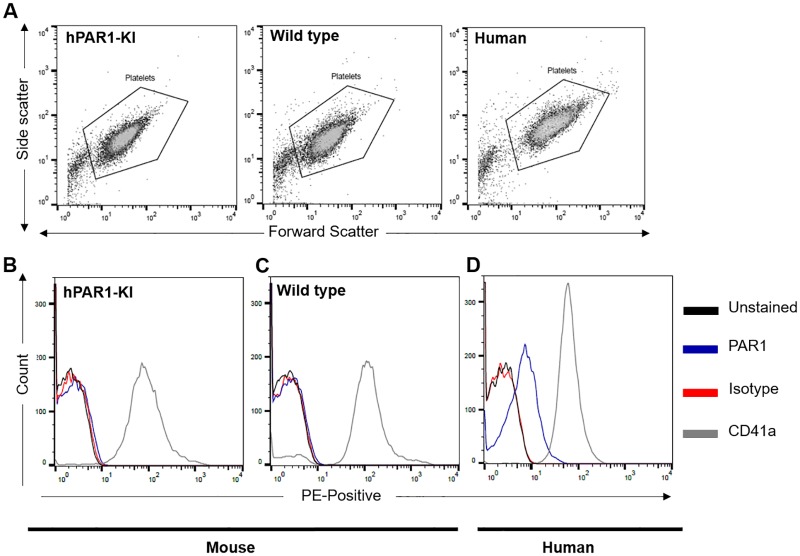
PAR1 is undetectable on the surface of platelets from hPAR1-KI mice. Platelets isolated from hPAR1-KI mice, wild type mice, or humans were either left unstained (black) or were incubated with a PE-conjugated antibody against one of PAR1 (blue) or CD41a (grey; positive platelet control), or an isotype control (red). **(A)** Platelets were gated by forward and side scatter as shown. **(B-D)** PAR1 was detected on human platelets but not platelets from either hPAR1-KI or wild type mice. Data shown are representative traces of n = 3 individual experiments.

We next examined PAR1 expression in platelets from hPAR1-KI mice. We were unable to detect PAR1 on the surface of platelets from hPAR1-KI mice by flow cytometry (Fig 3A). As with the functional experiments, platelets from wild type mice and humans served as effective negative and positive controls for PAR1 surface expression by this method, respectively (Fig 3B and 3C). In all experiments, the platelet specific marker, CD41, and normal forward/side scatter properties were used to confirm the integrity of the platelets being assessed (Fig 3).

**Fig 4:**
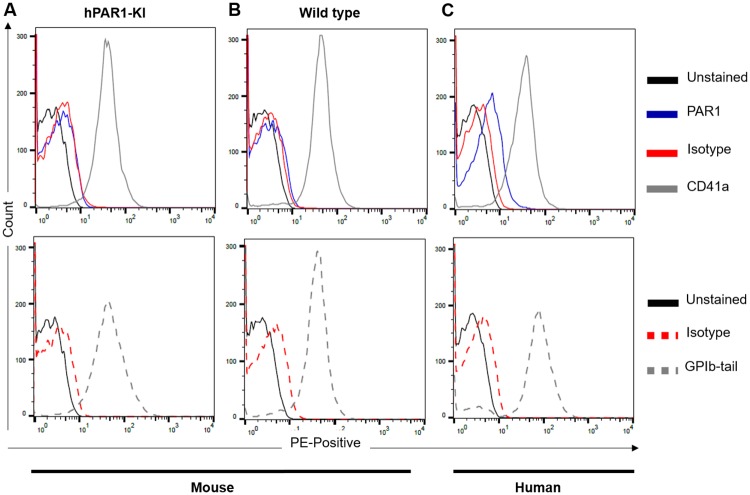
PAR1 is undetectable within platelets from hPAR1-KI mice. Platelets isolated from **(A)** hPAR1-KI mice, **(B)** wild type mice, or **(C)** humans were fixed and permeabilized prior to incubation with a PE-conjugated antibody against one of PAR1 (blue), CD41a (grey; positive control for platelets), isotype (red), or the cytoplasmic tail of GPIbα (grey dash; positive control for permeabilization). PAR1 was detected in human platelets but not in platelets from either hPAR1-KI or wild type mice. Data shown are representative traces of n = 3 individual experiments.

We also tested for PAR1 expression in permeabilized platelets. In these experiments, an antibody against the cytoplasmic tail of GPIbα [21] was used as a positive control for successful permeabilization. As predicted, this antibody only recognised an epitope on the internal surface of the platelet membrane, with no staining of intact platelets and strong staining in permeabilized platelets from both mice and humans (S4 Fig). However, we were unable to detect any PAR1 in permeabilized platelets from hPAR1-KI mice (Fig 4A). Again, platelet integrity was confirmed by CD41 expression in all cases, while permeabilization was confirmed by detection with the anti-GPIbα-tail antibody (Fig 4). Platelets from wild type mice and humans again served as controls (Fig 4B and 4C), indicating the anti-PAR1 antibody still binds its epitope in permeabilized cells.

**Fig 5:**
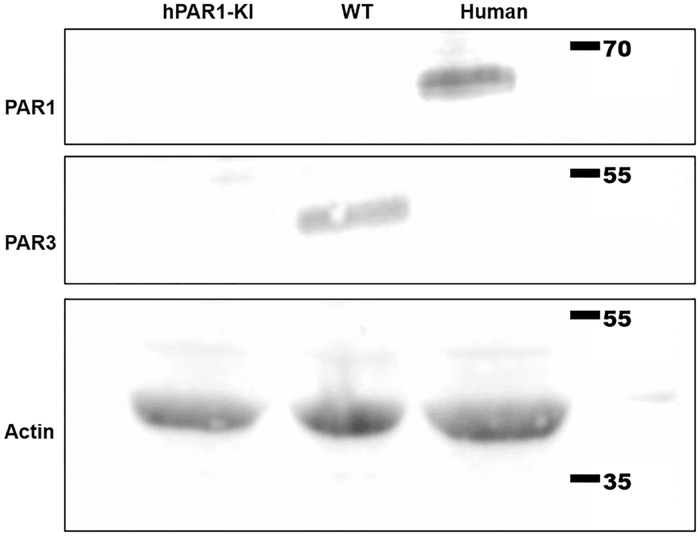
Protein for both PAR1 and Par3 is undetectable in platelets from hPAR1-KI mice. Whole cell lysates of platelets isolated from hPAR1-KI mice, wild type mice, or humans were probed for either PAR1 or Par3 protein by Western blot. PAR1 was readily detected in human platelets but not in platelets from wild type or hPAR1-KI mice. In the same samples, Par3 was detected in platelets from wild type mice but not in platelets from either hPAR1-KI mice or humans. Actin was used as a protein loading control for all lysates. Blots shown are representative of n = 3 experiments.

Finally, we also examined PAR1 expression by Western blot. Here, a band at the predicted size of approximately 75 kD was detected in lysates of human platelets, but not of wild-type or hPAR1-KI mouse platelets (Fig 5), mirroring the flow cytometry data. Together, these findings indicate PAR1 is not expressed at detectable levels in platelets from hPAR1-KI mice.

### PAR3 expression and function is disrupted in platelets from hPAR1-KI mice

Our genetic strategy is predicted to disrupt Par3 expression in platelets. This was confirmed genetically by PCR genotyping and sequencing (Fig 1). Regardless, given the lack of PAR1 expression or function in platelets from hPAR1-KI mice, we therefore also probed for Par3 protein in these platelets. We never detected Par3 expression by Western blot in human platelets, but routinely observed a band of the previously reported size (approximately 50kDa) in wild type mouse platelets (Fig 5). This band was absent in platelet lysates from all hPAR1-KI mice examined (Fig 5), further confirming disruption of *Par3* in hPAR1-KI mice. To further examine this, we tested for the predicted functional effects of *Par3*-deficiency.

**Fig 6:**
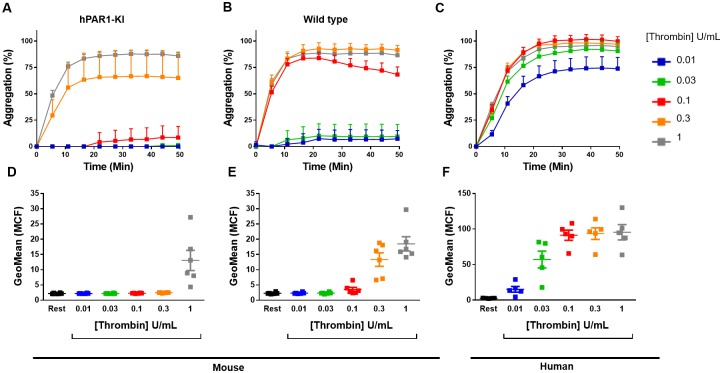
Platelets from hPAR1-KI mice exhibit diminished responses to thrombin. Platelets isolated from **(A,D)** hPAR1-KI mice, **(B,E)** wild type mice, or **(C,F)** humans were with thrombin and examined for **(A-C)** platelet aggregation by light transmission aggregometry or **(D-F)** P-selectin expression by flow cytometry. Note the approximate 3-fold decrease in sensitivity to thrombin-induced responses in platelets from hPAR1-KI mice versus wild-type mice in both assays. Data are mean ± SEM of n = 4–7 experiments.

Platelets from *Par3*-/- mice exhibit diminished responsiveness to thrombin [24]. Here, we observed a similar effect in platelets from hPAR1-KI mice. We examined platelet aggregation and P-selectin expression in response to thrombin. Platelets from hPAR1-KI mice exhibited an approximately 3-fold decrease in sensitivity to thrombin in both assays when compared to platelets from wild type mice (Fig 6). Specifically, aggregation of hPAR1-KI platelets was only achieved at thrombin concentrations of 0.3 U/mL and above (Fig 6A) compared with 0.1 U/mL and above for wild type platelets (Fig 6B). A similar pattern emerged when examining P-selection expression in response to thrombin: aggregation of hPAR1-KI platelets was only achieved at thrombin concentrations of 1 U/mL (Fig 6D) compared with 0.3 U/mL and above for wild type platelets (Fig 6E).

**Fig 7:**
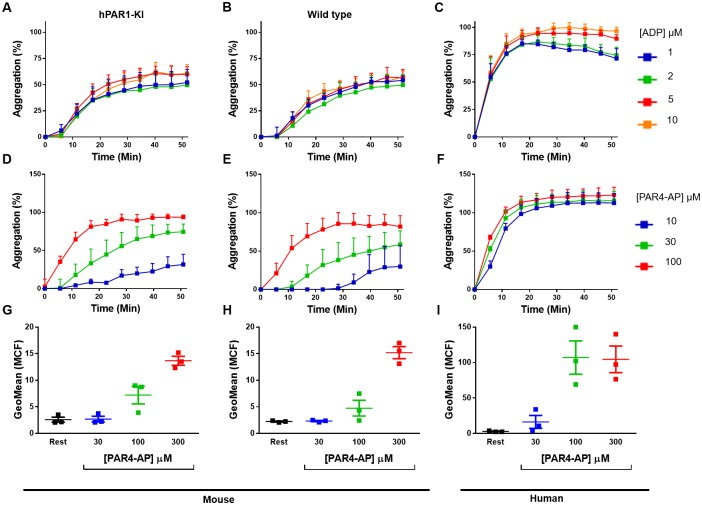
Platelets from hPAR1-KI mice respond normally to PAR1-independent platelet agonists. Platelets isolated from **(A,D,G)** hPAR1-KI mice, **(B,E,H)** wild type mice, or **(C,F,I)** humans were stimulated with either **(A-C)** ADP or **(D-I)** a PAR4-activating peptide (PAR4-AP) and assessed by **(A-F)** platelet aggregation or **(G-I)** P-selectin expression. Note the near-identical concentration-dependent responses in platelets from hPAR1-KI and wild type mice in all cases examined. Data are mean ± SEM of n = 3 experiments.

Importantly, hPAR1-KI platelets responded normally to other agonists, PAR4-AP and ADP (Fig 7). When comparing platelets from hPAR1-KI and wild type mice, near-identical concentration-responses were observed for ADP-induced aggregation (Fig 7A and 7B), PAR4-AP-induced aggregation (Fig 7D and 7E), and PAR4-AP-induced P-selectin expression (Fig 7G and 7H). These functional data are consistent with the phenotype observed in platelets from *Par3-/-* mice and, when combined with the failure to detect Par3 expression in hPAR1-KI platelets, suggest correct targeting and *Par3* deletion in hPAR1-KI mice.

## Discussion

PARs are leading targets for new anti-platelet drugs, exemplified by the recent approval of the PAR1 antagonist, vorapaxar, and the early clinical development of the PAR4 antagonist, BMS-986120. Given the increased focus of examining PAR antagonists as anti-platelet agents, there is much interest in developing an appropriate small animal model for *in vivo* testing of such drugs. This has proved to be a major limitation in the field, as the PAR1/PAR4 expression profile of human platelets is only shared amongst primates. Here, we attempted to create a genetically-modified mouse that mimics the PAR1/PAR4 expression profile of human platelets. To do this, we knocked the human *PAR1* gene in to the mouse *Par3* locus in a manner that provided for platelet-specific replacement of Par3 with PAR1. Correct targeting was confirmed genetically and was apparent functionally. Yet despite this, we were unable to detect expression or function of PAR1 in platelets from these mice, indicating the approach used here is insufficient to ‘humanize’ platelet PAR expression in mice by inducing PAR1 expression in place of Par3.

Forced PAR1 expression has been previously achieved using transgene-based approaches in other cell types. For example, mouse PAR1 (Par1) was specifically expressed in endothelial cells via the Tie2 promoter/enhancer [25] and in cardiomyocytes via the αMHC promoter [26]. In addition, PAR1 has been expressed in mouse mammary gland epithelium [27]. In contrast to these successes, a previous attempt failed to express either Par1 or PAR1 transgenes in mouse platelets via multiple platelet-specific promoters [18]. The reasons for the inability to express PAR1 in mouse platelets remain unknown. However, given the limitations and lack of control associated with transgene-based approaches, we used a knock-in approach in which *PAR1* was targeted to the *Par3* locus, essentially resulting in replacement of Par3 with PAR1, and with expression governed by the endogenous mouse *Par3* promoter.

Correct genetic targeting was confirmed by multiple methods, including Southern blot analysis of founders, PCR genotyping across the insert, and PCR analysis of correct Pf4-Cre-mediated excision in the DNA in isolated megakaryocytes. Correct targeting was also apparent by the absence of Par3 expression in platelets from hPAR1-KI mice. The disruption of Par3 expression was further supported by functional analyses of these platelets that revealed a phenotype remarkably similar to that observed in platelets from *Par3*^-/-^ mice [24], with an approximately 3-fold decrease in sensitivity to thrombin-induced platelet activation. Together, this detailed analysis indicates correct targeting occurred in these mice and resulted in disruption of Par3 expression. However, no replacement PAR1 expression or function was detected.

The reason behind this lack of expression of PAR1 in mouse platelets is unknown. Our initial functional studies showed that hPAR1-KI platelets did not respond to a PAR1-specific agonist but responded normally to other agonists, suggesting that the receptor was either absent or unable to signal. We probed extensively for protein expression but were unable to detect PAR1 in platelets from hPAR1-KI mice via flow cytometry or Western blot. The lack of detection of PAR1 even in permeabilizsed platelets (by flow cytometry) or in whole cell platelet lysates (by Western blot) strongly suggest that the PAR1 protein is either not translated or is very rapidly degraded. The inability of mouse platelets to ectopically express PAR1 so far appears unique, but remains unexplained and could arise from a number of critical stages in the development of mature PAR1 protein. While possible that the Par3 signal peptide is insufficient to guide PAR1 to the cell surface [28], the highly conserved nature of signal peptides coupled with our observation of robust surface expression of the fusion protein in HEK293T cells suggest otherwise. Other potential explanations include as yet unidentified mechanisms that appear unique to the mouse platelet, such as poor or defective N-linked glycosylation of the receptor leading to receptor instability and signaling dysfunction [29, 30]. Since the primary aim of this study was to generate a research tool for *in vivo* thrombosis studies, we did not explore the precise mechanism behind the lack of tolerance for PAR1 expression in mouse platelets. Whether or not replacement of the entire human *PAR1* gene in the mouse *Par3* locus will be sufficient to drive expression in mouse platelets remains unknown. Regardless, it appears that other approaches will need to be examined if a non-primate model is to be used for pharmacological screening of drugs targeting platelet PARs.

## Supporting Information

S1 Fig The receptor generated in hPAR1-KI mice can be expressed in HEK293T cells. The fusion protein predicted to be expressed by hPAR1-KI mice, consisting of the mPAR3 signal peptide attached to the mature hPAR1 receptor, can be expressed on the surface of transfected HEK293T cells. HEK293T cells were transfected with native hPAR1 (purple), the mPAR3/hPAR1 fusion (pink), or empty vector (yellow). Note the similar level of detectable expression of native hPAR1 and the mPAR3/hPAR1 fusion. Also shown is a positive control for surface expression (Thy-1) in cells transfected with the empty vector.
(TIFF)

S2 Fig Southern blot analyses of targeted ES cells and founder mice. **(A)** ES cell clones were screened for targeting by Southern blot. Targeted clones are indicated by the expected additional band at 8.3kb (e.g. clones D1 and D4). **(B)** Founder mice screened by the same Southern blot approach as in (A), showing results for 13 mice from two litters. Seven of the 13 mice genotyped as heterozygous targeted, exhibiting both the wt (11.6 kb) and mutant band (8.3kb).
(TIFF)

S3 Fig Sequence annotation for the hPAR1-KI allele. **(A)** Predicted genomic DNA sequence for the hPAR1-KI allele, showing *Par3* exon 1 highlighted in grey, the position of primers 3F and 4R in red, the loxP site in yellow, and *PAR1* exon 2 in blue. Red text indicates the sequence output of the PCR product from primers 3F and 4R, which was identical to the predicted genomic DNA sequence. **(B)** Annotated sequence of the predicted coding sequence and protein chimera, comprising the Par3 signal peptide (grey text) and PAR1 mature receptor (blue text). Red text indicates sequenced PCR product amplified from genomic DNA of hPAR1-KI mice, aligning to the predicted region of PAR1 coding sequence.
(TIFF)

S4 Fig Confirmation of platelet permeabilization. Platelets isolated from a mouse (left) or human (right) were either left intact (top) or were permeabilized with saponin (0.1%; bottom) prior to incubation with an antibody against the intracellular C-terminal of GPIbα (grey) or isotype control (red). The GPIbα tail antibody produced a rightward shift over isotype only in permeabilized platelets of both species, confirming successful permeabilization. Data shown are representative traces of n = 3 individual experiments.
(TIFF)

S1 Table Whole blood cell counts from wild type and hPAR1-KI mice. (DOCX)
