# Supplementary figures and images for: Humanizing the Protease-Activated Receptor (PAR) Expression Profile in Mouse Platelets by Knocking PAR1 into the Par3 Locus Reveals PAR1 Expression Is Not Tolerated in Mouse Platelets
Source: PLoS One. 2016 Oct 27;11(10):e0165565. doi: 10.1371/journal.pone.0165565 (PMC5082849; doi:10.1371/journal.pone.0165565)

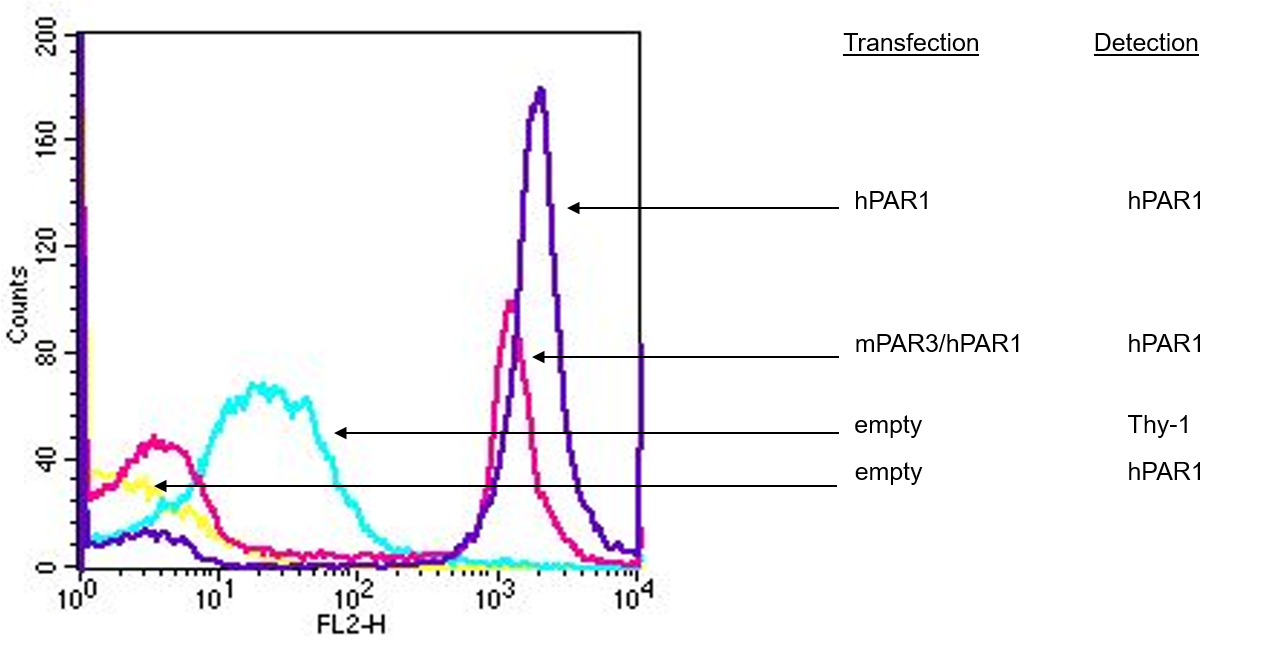

Supplement: S1 Fig — The fusion protein predicted to be expressed by hPAR1-KI mice, consisting of the mPAR3 signal peptide attached to the mature hPAR1 receptor, can be expressed on the surface of transfected HEK293T cells. HEK293T cells were transfected with native hPAR1 (purple), the mPAR3/hPAR1 fusion (pink), or empty vector (yellow). Note the similar level of detectable expression of native hPAR1 and the mPAR3/hPAR1 fusion. Also shown is a positive control for surface expression (Thy-1) in cells transfected with the empty vector. (TIFF) [file pone.0165565.s001.tiff]

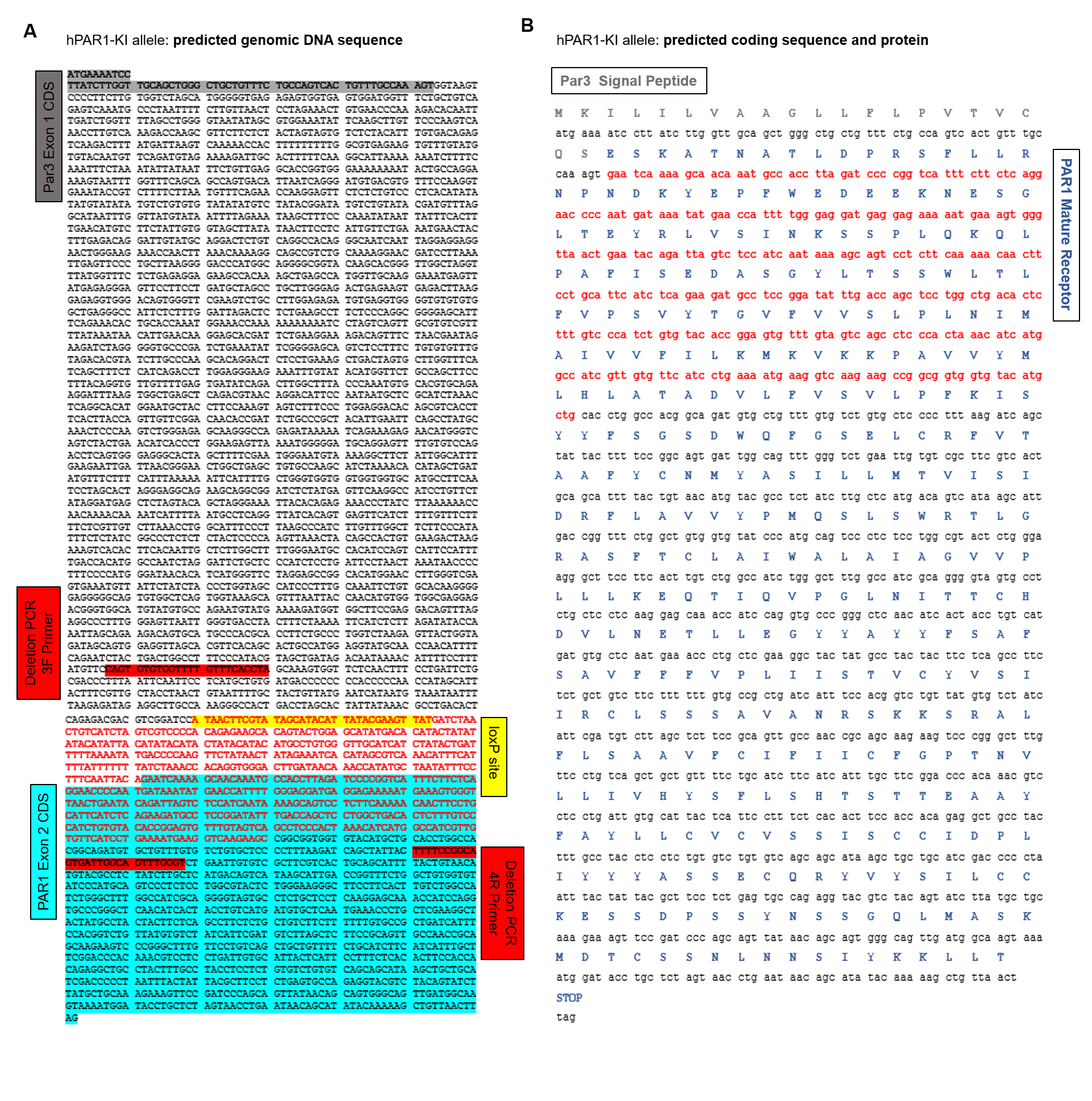

Supplement: S3 Fig — (A) Predicted genomic DNA sequence for the hPAR1-KI allele, showing Par3 exon 1 highlighted in grey, the position of primers 3F and 4R in red, the loxP site in yellow, and PAR1 exon 2 in blue. Red text indicates the sequence output of the PCR product from primers 3F and 4R, which was identical to the predicted genomic DNA sequence. (B) Annotated sequence of the predicted coding sequence and protein chimera, comprising the Par3 signal peptide (grey text) and PAR1 mature receptor (blue text). Red text indicates sequenced PCR product amplified from genomic DNA of hPAR1-KI mice, aligning to the predicted region of PAR1 coding sequence. (TIFF) [file pone.0165565.s003.tiff]

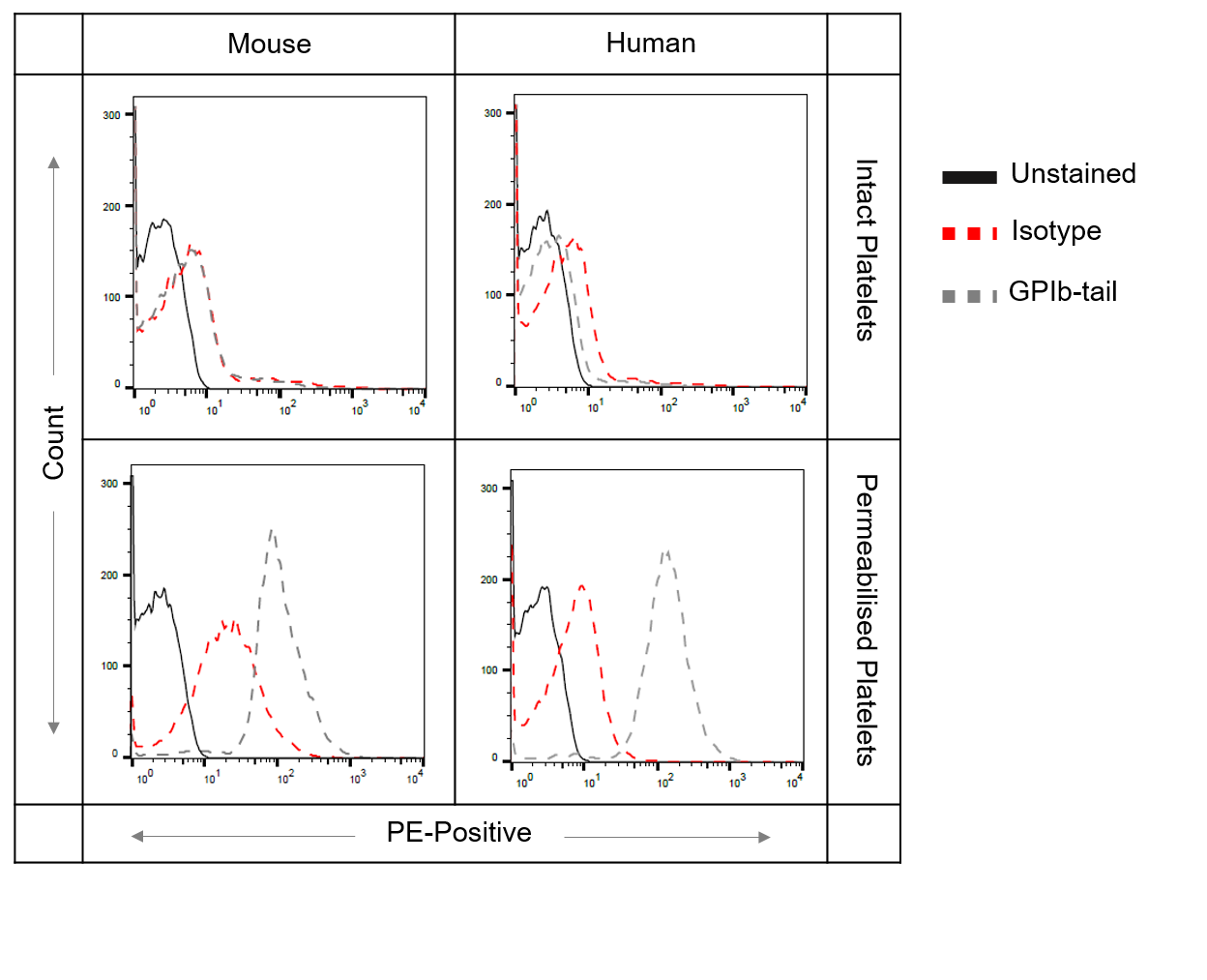

Supplement: S4 Fig — Platelets isolated from a mouse (left) or human (right) were either left intact (top) or were permeabilized with saponin (0.1%; bottom) prior to incubation with an antibody against the intracellular C-terminal of GPIbα (grey) or isotype control (red). The GPIbα tail antibody produced a rightward shift over isotype only in permeabilized platelets of both species, confirming successful permeabilization. Data shown are representative traces of n = 3 individual experiments. (TIFF) [file pone.0165565.s004.tiff]
