# Supplementary material for: Humanizing the Protease-Activated Receptor (PAR) Expression Profile in Mouse Platelets by Knocking PAR1 into the Par3 Locus Reveals PAR1 Expression Is Not Tolerated in Mouse Platelets
Source: PLoS One. 2016 Oct 27;11(10):e0165565. doi: 10.1371/journal.pone.0165565 (PMC5082849; doi:10.1371/journal.pone.0165565)
Supplement: S1 Table — (DOCX) [file pone.0165565.s005.docx]

**S1 Table. Whole blood cell counts from wild type and hPAR1-KI mice.**

|  | Wild type | hPAR1-KI |
| --- | --- | --- |
| WBC (10^3^/μL) | 7.3 ± 3.2 | 7.1 ± 2.9 |
| RBC (10^6^/μL) | 9.4 ± 1.2 | 9.6 ± 0.7 |
| PLT (10^3^/μL) | 887 ± 50 | 888 ± 98 |

WBC = white blood cells, RBC = red blood cells, PLT = platelets.
